# Healthcare Professionals’ Learning Needs and Perspectives on Essential Information in Genetic Cancer Care: A Systematic Review

**DOI:** 10.3390/cancers16111963

**Published:** 2024-05-22

**Authors:** Sun-Young Park, Youlim Kim, Maria C. Katapodi, Yeon-Joo Kim, Heejung Chae, Yoon-Jung Choi, Kum Hei Ryu, Eun-Gyeong Lee, Sun-Young Kong, So-Youn Jung

**Affiliations:** 1 College of Nursing, Daegu Catholic University, Daegu 42472, Republic of Korea; psy23@cu.ac.kr; 2 College of Nursing, Kosin University, Busan 49104, Republic of Korea; goshimak@naver.com; 3 Department of Clinical Research, University of Basel, 4055 Basel, Switzerland; maria.katapodi@unibas.ch; 4 Department of Radiation Oncology, National Cancer Center, Goyang 10408, Republic of Korea; yjkim1785@ncc.re.kr; 5 Center for Breast Cancer, National Cancer Center, Goyang 10408, Republic of Korea; hchae21@ncc.re.kr (H.C.); bnf333@ncc.re.kr (E.-G.L.); 6 Department of Cancer Control & Population Science, National Cancer Center Graduate School of Cancer Science and Policy, Goyang 10408, Republic of Korea; yj_choi@ncc.re.kr (Y.-J.C.); kumheiryu@ncc.re.kr (K.H.R.); 7 National Cancer Control Institute, National Cancer Center, Goyang 10408, Republic of Korea; 8 Center for Cancer Prevention & Detection, National Cancer Center, Goyang 10408, Republic of Korea; 9 Department of Laboratory Medicine & Genetic Counseling Clinic, National Cancer Center, Goyang 10408, Republic of Korea

**Keywords:** hereditary cancer syndromes, genetic services, healthcare professionals, educational needs, information, systematic review

## Abstract

**Simple Summary:**

Increasing demand for genetic testing and counseling among families with hereditary cancers has drawn attention to the genetic skills and knowledge of healthcare professionals (HCPs). However, many HCPs face challenges regarding confidence in communicating genetic risk to their patients and accessing genetic training programs. Developing genomic educational strategies and standardizing the curriculum for HCPs is critical to improving genetic care. This systematic review identified the learning needs of HCPs and compared them across professions, along with their perspectives on essential information for families affected by hereditary cancer. While HCPs recognized the importance of providing a wide range of information to families affected by hereditary cancer and emphasized enhancing practical counseling skills, their learning needs varied by profession. Our findings have implications for developing training programs for HCPs, underscoring the importance of developing targeted training programs and resources aligned with their specific profession.

**Abstract:**

Background: The increased demand for genetic testing and counseling necessitates healthcare professionals (HCPs) to improve their genetic competency through training programs. This systematic review identified HCPs’ learning needs and their perspectives on essential information for families with hereditary cancer. Methods: This review covered studies published from 2013 to 2024 across five databases. Data were analyzed using a content analysis. Results: Thirteen studies involving 332 HCPs were analyzed. Most studies focused on the learning needs of physicians caring for families affected by Hereditary Breast and Ovarian Cancer in North America and Europe. HCPs required training emphasizing practical counseling skills over the basics of genetics. Learning needs varied by profession: physicians needed training in assessing cancer risk and supporting decision-making in risk management; nurses required information on resources and the genetic care system; genetic counselors sought guidance on family communication and planning. Essential information identified for families included risk-reducing strategies, personalized cancer risk assessment, family implications, psychological issues, (cascade) genetic testing, and social concerns. Conclusions: The findings have implications for the development of training programs for HCPs, emphasizing the need for tailored training based on professions. Future research should explore the needs of HCPs caring for families with diverse hereditary cancers and cultural backgrounds.

## 1. Introduction

The rising demand for cancer risk assessments and genetic testing, and the increasing prevalence of hereditary cancers have led to a surge in the need for genetic counseling [1,2]. Genetic counseling plays a crucial role in improving the physical, psychological, and social well-being of families affected by hereditary cancer [3]. Healthcare professionals (HCPs), including physicians, nurses, and genetic counselors, are integral to the entire process of genetic care, including risk assessment, management, and counseling for hereditary cancer [4,5]. Effective risk management and risk communication rely heavily on HCPs’ genetic knowledge and skills, which enhance patient and family access to information [6] and enable informed decision-making for families affected by hereditary cancer [4].

The development of genomic learning standards and education strategies for HCPs have gained significant attention [7,8,9]. However, HCPs often face challenges in managing complex cancer risks and providing effective counseling due to their limited access to specialized training programs [10]. Additionally, HCPs often experience confidence issues when communicating genetic risk, particularly when dealing with patients with limited health literacy, and when managing patients with variants of uncertain significance (VUS) in a clinical setting [11]. The lack of genetic knowledge and communication skills among HCPs hinders the effective sharing of genetic information with families affected by hereditary forms of cancer [2].

Given the importance of HCPs’ perspectives in developing relevant educational programs [12], it is essential to clearly define the specific knowledge and competencies required for HCPs to provide effective genetic counseling [13], ensuring that HCPs have the appropriate skills and resources to support their patients [12]. This systematic review aimed to identify HCPs’ learning needs allow them to provide genetic care to families with hereditary cancer, along with their perspectives on what constitutes essential information for these families. The review sought to identify and appraise the existing data to inform the development of targeted training programs and educational strategies that enhance HCPs’ genetic counseling abilities and knowledge to address their unique needs and effectively support families with hereditary forms of cancer.

## 2. Methods

This systematic review followed Sandelowski’s mixed-method review methodology [14] to explore how the topic was presented in various study designs, including studies that collected quantitative and/or qualitative data. The review was conducted and written according to the Preferred Reporting Items for Systematic Reviews and Meta-Analysis (PRISMA) checklist, as outlined by Page et al. (2021) [15]. The protocol for this review was registered on the PROSPERO website (registered code: CRD42023464637).

### 2.1. Eligibility Criteria

The inclusion and exclusion criteria for article selection are outlined in Table S1. Briefly, studies were eligible if they were conducted among HCPs, such as physicians, nurses, genetic counselors, and clinical geneticists, who treat or counsel individuals at risk of or with hereditary forms of cancer. We included articles that reported HCPs’ learning needs related to genetic care or counseling for families with hereditary cancer and HCPs’ perspectives on what constitutes essential information for these families. Studies employing quantitative, qualitative, and mixed methods approaches were included, whereas original research articles, such as reviews, letters, and editorials, were excluded. We included studies published in English from 2013 to ensure the inclusion of up-to-date evidence. The first search was conducted in May 2023, with the final search updated in April 2024.

### 2.2. Search Strategy

In collaboration with the research team, a literature search professional (Y.-J. Kim) with 6 years of experience conducted a comprehensive literature search across MEDLINE, EMBASE, CINAHL, the Cochrane Central Register of Controlled Trials, and PsycINFO. To optimize the search strategy, keywords related to target populations and research interests were combined using Boolean operators (e.g., hereditary cancer AND healthcare professionals AND (learning needs OR perspective)). Specific search terms and strategies are detailed in Table S2.

**Figure 1:**
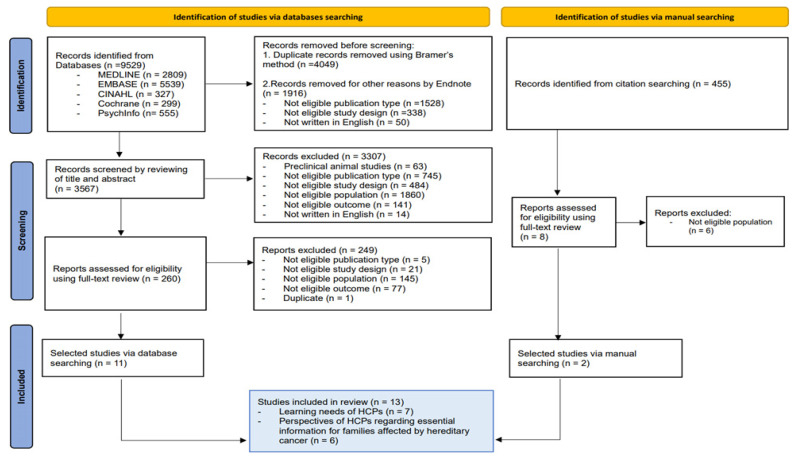
PRISMA 2020 flow diagram.

All citations and abstracts identified by the search strategy were exported to the reference-management software (EndNote 21, Clarivate Analytics, Philadelphia, PA, USA). Additionally, we manually searched and reviewed references from the selected articles to identify additional articles that corresponded to the inclusion criteria using Google Scholar. Duplicates were removed following Bramer’s method [16]. After the initial literature search, two reviewers (S.-Y. Park, Y Kim) screened, in blinding mode, titles and abstracts for relevance based on the inclusion/exclusion criteria, and compared their screening results. In cases of disagreement between reviewers, if at least one reviewer identified that article may potentially meet the inclusion criteria, a third reviewer searched and assessed the full text of the article. Upon reviewing the full text, those deemed not to meet the inclusion criteria were excluded. Any disagreements between reviewers were resolved through discussion in the research team. The reasons for exclusion are recorded in Table S3. The PRISMA flow diagram shows the study selection process (Figure 1).

### 2.3. Assessment of Methodological Quality

Two reviewers (S.-Y. Park, Y Kim) independently assessed the methodological quality of the included studies using the Mixed Methods Appraisal Tool (MMAT) [17]. This tool is specifically designed to assess studies across different methodologies, including qualitative, quantitative (e.g., randomized controlled trials, non-randomized studies, and descriptive studies), and mixed methods studies.

In the assessment process, two key screening questions from the MMAT tool were applied to each study: one focused on the clarity of the research question, while the other assessed the appropriateness of the collected data in addressing the research question. If the study failed to meet at least one of these two key screening criteria, it was excluded from further review. The MMAT includes five questions for each study design. The outcomes of these appraisals were categorized as “yes”, “no”, or “can’t tell”, reflecting the assessed methodological quality of each study. Discrepancies in the quality assessment conducted by the reviewers were resolved through discussions during team meetings. Details of the assessment outcomes can be found in Table S4.

### 2.4. Data Extraction

We extracted data using two key components. Initially, one reviewer (S.-Y. Park) extracted key characteristics of the included studies using a specific data collection form provided in Table S5. This form was designed following a comprehensive review of the related literature to determine the needs of HCPs and their perspectives on essential information in genetic care. It captured critical information from each study, including the first author, year of publication, country, study design, data collection method, number of participants, study aim, targeted patients and families of HCPs, and research interest.

The second component focused on extracting and synthesizing data that revealed HCPs’ perceptions of what constituted essential information for families affected by hereditary forms of cancer, using Sandelowsky’s approach to integrate the different types of data (e.g., qualitative- and quantitative data) [14]. Based on the Bayesian method suggested by Pearson (2015) [18], we converted quantitative data into a format suitable for qualitative analysis, allowing for the seamless integration of quantitative and qualitative data and a comparison between the extracted data within the analysis framework.

### 2.5. Data Analysis

We employed a deductive content analysis to analyze the extracted data, as outlined by Elo and Kyngas (2008) [19]. The analysis involved synthesizing the findings into a set of statements that effectively represented the aggregated data, focusing on the categorization of findings based on their similarities. In the first step, one team member (S.-Y. Park) developed an abstraction tool, which utilized a selection of five randomly chose studies [1,10,20,21,22]. In developing the abstraction tool, we extracted data line-by-line from each study, coded them to label the learning needs and perspectives of HCPs, and classified similar codes into the same categories. Following pilot testing and the subsequent refinement of this abstraction tool, two reviewers (S.-Y. Park, Y. Kim) independently extracted, coded, and categorized the learning needs and perspectives of HCPs from various studies using MAXQDA 2020 software (VERBI GmbH, Berlin, Germany) [19]. New codes that did not align with the pre-established coding list were assigned to a new category, as suggested by Hsieh and Shannon (2005) [23]. Any differences in coding between the reviewers were resolved through discussion [19].

## 3. Results

### 3.1. Selection of Studies

Initially, we identified 9529 articles after searching all databases (Figure 1). After eliminating 4049 duplicates and 1916 articles that did not meet the inclusion criteria (not an eligible publication type or study design, or written in English) by screening using Endnote, we reviewed 3567 articles based on their titles and abstracts, with 7.2% (n = 257 articles) of the results leading to a disagreement between the two reviewers. This first selection stage yielded 260 potentially relevant studies. After conducting a full-text review of these studies, we included 11 studies [1,10,11,20,21,24,25,26,27,28,29]. A manual search through the references of these selected articles identified an additional 455 studies. Among these, nine underwent full-text review, leading to the inclusion of two more studies [22,30]. Consequently, this systematic review included a total of 13 studies.

### 3.2. Characteristics of Included Studies

Table 1 summarizes the included studies. Among the 13 included studies, a greater proportion (61.5%) was published after 2019 [1,11,20,21,22,27,28,29] compared to the period between 2013 and 2018 [10,24,25,26,30]. Geographically, most studies were conducted in North America (46.2%) [10,21,24,26,28,29] and Europe (38.5%) [1,20,25,27,30], followed by Australia [22] and Malaysia [31]. In terms of the study design, there were seven qualitative studies [10,20,25,26,27,28,29], four quantitative descriptive studies [1,11,24,30], and two mixed methods studies [21,22].

The selected studies included a total of 332 HCPs (range = 6–111 HCPs, mean ± SD = 25.5 ± 31.5). Most HCPs whose learning needs and essential information for patients were assessed were physicians (n = 205) and genetic counselors (n = 86), followed by clinical geneticists (n = 13) and nurses (n = 12) (Table 1). Over half of the studies explored various mixed professions’ learning needs and their perspectives on essential information for patients (7/13 studies) [21,22,24,26,27,29,30]; the other half examined a single profession, such as physicians (three studies) [8,12,31], nurses (one study) [20], clinical geneticists (one study) [25], and genetic counselors (one study) [10] (Table S5). Most studies (85%) reported the perspectives of HCPs who cared for individuals affected by hereditary breast and ovarian cancer (HBOC) [1,10,11,20,21,22,24,26,28,29,30], followed by one study focusing on Lynch syndrome [27], and one study focusing on various hereditary cancer syndromes [25]. Nine studies [1,10,11,20,22,24,25,28,29] examined the learning needs of HCPs regarding risk management and counseling for families with hereditary cancer (Table 2); eight studies [1,10,11,20,24,25,28,29] compared HCPs’ learning needs according to their profession (Table 2); and six studies [10,21,22,26,27,30] explored the perspectives of HCPs on information that is essential for families with hereditary forms of cancer (Table 3).

### 3.3. Methodological Quality

Table S4 presents the results of the methodological quality appraisal conducted on the eligible studies. The number of “yes” responses to each question ranged from 1 (representing 20% compliance) to 5 (indicating 100% compliance). Among these studies, 70% [10,11,20,22,25,27,28,29,30] were identified as having a low risk of bias, since each received a ‘yes’ in all five categories. Additionally, 30% of the studies [1,21,24,26] were categorized as having a moderate risk of bias, with at least 50% of the criteria being met.

### 3.4. Learning Needs of HCPs in Genetic Care

Table 2 provides the learning needs of HCPs regarding providing genetic care to families affected by hereditary cancer. Nine studies [1,10,11,20,22,24,25,28,29] highlighted that HCPs require training in five distinct areas, encompassing 19 subcategories.

*1. Methods for supporting decision-making:* In five studies [1,20,24,28,32], HCPs emphasized the need for training to improve their practical knowledge and skills, enabling them to support the decision-making process for cancer risk management. HCPs expressed a keen interest in understanding patients’ needs related to decision-making [1], psychosocial challenges [1], and coping strategies [20,28]. HCPs recognized the importance of training in facilitating patient involvement and shared decision-making [1,24] and utilizing methods to clarify their values [20]. HCPs expressed the need for guidance in the decision-making process within their healthcare centers (e.g., who initiates the referral for genetic evaluation or suggests genetic testing, and how to communicate genetic information) [20] and the optimal time for help with decisions [25]. Given the importance of personalized care in managing hereditary cancer risk, HCPs required training on how to integrate and apply various patient-specific factors in their decision-making for risk-reduction strategies or genetic testing [28]. These factors include age, comprehension, adherence, the type of genetic predisposition, existing cancers, financial situation, life stage, comorbidities, smoking habits, and anxiety levels [28].

*2. Methods for assessing and managing cancer risk:* HCPs sought information on, and an increase in the availability of, predictive models specific to hereditary cancer [1,24]. These models are valuable for recommending appropriate clinical management for patients, and for enabling informed decision-making based on calculated risks of cancer occurrence or recurrence [24]. HCPs expressed a need to enhance their understanding of the genetic testing process, including the available options for genetic testing among different members of families with hereditary cancer [1], the process of ordering these tests [1], and related costs [11]. Importantly, HCPs emphasized the need for training in interpreting genetic testing results [1,20] and comprehending the clinical implications of these results [1]. HCPs required a deeper understanding of VUS, since insufficient evidence makes assessing cancer risk and recommending preventive options challenging [1].

*3. Resources for supporting hereditary cancer families:* HCPs reported the need for both material and human resources to support families with hereditary cancer [11,20,32]. Regarding material resources, HCPs considered decision aids [25], patient leaflets [11], and visual aids (e.g., videos containing the real-life experiences of similar families) to be valuable tools for enhancing patient understanding during genetic counseling [25,29]. In terms of human resources, HCPs expressed a desire to know whether their clinics have genetic counselors or genetic nurses [11,20].

*4. Guidance for supporting familial issues:* HCPs required the knowledge and skills necessary to assist families coping with the results of genetic testing [10,22,29], as they can significantly impact family dynamics and physical and psychological well-being [29]. They also required training when communicating with different family members [10,29], particularly in situations of patient-mediated disclosure of genetic test results [22]. Additionally, they required information regarding guiding decision-making for family planning, including different fertility options after surgical menopause [10], and managing familial distress [10].

*5. The role of profession:* HCPs expressed a keen interest in clarifying their specific roles in the care of families with hereditary cancer. Specifically, primary care providers, such as general practitioners, sought clarity on their role in identifying individuals who need genetic testing [24], while nurses were interested in defining their role in collaborating with inter-professional teams [20].

### 3.5. Comparison of Learning Needs according to Profession

Table 2 summarizes the distinct learning needs of both non-genetic professionals and genetic professionals. We identified five studies focusing on non-genetic professionals [1,11,20,24,28] and two studies focusing on genetic professionals [10,25].

Non-genetic professionals, such as physicians and nurses, required a wider range of knowledge compared to genetic professionals such as genetic counselors and clinical geneticists [1,20,24,25,28]. Physicians specifically sought information regarding the decision-making needs of families with hereditary cancer [1,20,28], methods for shared decision-making [1,24], factors to consider when recommending clinical management [28], and methods for assessing and managing cancer risk, including predictive models, cancer risk-reducing strategies, the process and clinical interpretation of genetic testing, and VUS [1,11,20,24]. They also required information regarding resources for supporting families with hereditary cancer [11,20,25] and an understanding of their professional role in this area [24]. Nurses, on the other hand, sought information about ways to clarify values [20], the decision-making processes within their centers [20], the clinical interpretation of genetic testing [20], and their role in genetic counseling and education [20]. Genetic professionals, such as genetic counselors and clinical geneticist, required a narrower range of education compared to non-genetic professionals, as they did not report learning needs regarding the methods for supporting decision-making and assessing/managing cancer risk. Genetic counselors sought guidance in supporting families with hereditary cancer [10,29], and clinical geneticists required information regarding the optimal time to provide aid with decisions [25] and material resources [25].

### 3.6. Information for Hereditary Cancer Families Recommended by HCPs

Six studies [10,21,22,26,27,30] highlighted HCPs’ perceptions of what is essential information for families with hereditary cancer. This could be divided into six distinct categories, including risk-reducing strategies, personalized risk assessment, family implications, psychological issues, genetic testing, and social issues (Table 3).

*1. Cancer risk-reducing strategies:* HCPs in four studies emphasized the need for families affected by hereditary cancer to receive comprehensive information on risk-management strategies [10,21,22,30]. This includes detailed descriptions of various strategies for risk-reducing surgeries, screening, and chemoprevention, covering aspects such as the process and timing of, and access to, these services [10,21,22,30]. HCPs also emphasized the importance of understanding the impact of each risk management strategy, including the side effects, potential cancer risks associated with each strategy, changes in body image and breastfeeding, lifestyle modifications, and financial implications [10,22]. Finally, HCPs considered it essential to provide information regarding insurance coverage for each strategy [22].

*2. Personalized cancer risk:* HCPs stressed the necessity of delivering personalized information to families with hereditary cancer [10,22,27,30], covering various aspects, such as the timing of the cancer’s development [10,22], the lifetime risk of cancer occurrence and recurrence [22], the likelihood of specific cancer types occurring based on age and family history [27], and cancer risks for men with certain genetic variants [30].

*3. Family implications of hereditary cancer risk:* HCPs recognized that families affected by hereditary cancer require information about the familial implications [10,22,30], including how to communicate genetic test results to relatives [10,22], and reproductive and family planning issues (e.g., available fertility options after receiving cancer risk-reduction strategies, and considerations for contraception) [10,22]. HCPs noted the importance of informing families about the modes by which the pathogenic variant could be transmitted to children, grandchildren, and other relatives [22,30].

*4. Psychological issues:* According to two articles, HCPs stressed the importance of providing psychological support for individuals and families coping with the challenges associated with hereditary cancer [10,22]. To address both individual (e.g., anxiety, fear, worry, and uncertainty) and family distress (e.g., guilt, worry about family members’ cancer risk or progression), HCPs recommended providing information on emotional management and other coping strategies [22], as well as affective forecasting of the trajectory of hereditary cancer risk [10].

*5. Genetic testing*: In two articles, HCPs emphasized the significance of offering detailed information regarding cascade genetic testing to relatives in families harboring pathogenic variants [10,22], including both the benefits and potential risks associated with genetic test results [10,22], the costs associated with cascade testing [22], how to understand test results such as gene status [22], and the optimal timing for genetic testing [10].

*6. Social issues related to genetic testing*: In two studies, HCPs underscored the importance of providing comprehensive information on various social dimensions to families with hereditary cancer [10,22]. This information includes how to cope with the stigma associated with a genetic diagnosis [22], how to disclose a genetic diagnosis to friends [22], future health insurance issues [10], and access to resources for social support such as groups and networks [10].

## 4. Discussion

This systematic review comprehensively summarized data from 13 studies regarding the learning needs of HCPs and their perspectives on the essential information required by families affected by hereditary cancer. Most studies were conducted in North America and Europe, mainly reporting the perception of physicians caring for HBOC-affected families. HCPs require training that can enhance their practical skills and their knowledge about the resources that can support families with hereditary cancer, focusing on decision-making, cancer risk assessment and management, and the roles of each profession. Learning needs vary by profession, with non-genetic professionals requiring a wider range of training compared to genetic professionals. HCPs perceived essential information for these families to focus on risk-reducing strategies, personalized risk assessment, family implications, psychological issues, genetic testing, and social issues. Our results provide evidence-based insights for developing educational programs for HCPs and genetic counseling for families, suggesting the need for tailored educational approaches for different HCPs and specific genetic counseling content.

Notably, the typology developed in this review might have implications for the development of training programs for HCPs in genetic cancer care, enhancing the relevance and effectiveness of educational programs. This typology differs from previous reviews, which explored the gaps in the genetic knowledge of HCPs based on their status and gaps in their skills and knowledge [2]. In this review, data were systematically extracted from direct reports of HCPs, regardless of their profession, making this typology more comprehensive and providing a structured framework for educational materials. Specifically, our findings can guide the development of tools for assessing learning needs in developing the content and structure of complementary learning sources, such as interactive webinars and online courses, which can provide HCPs with training based on their own needs. This might enable HCPs to improve their knowledge and skills in genetic care, customizing their learning according to their specific professional needs and field of practice [9].

Our findings indicate HCPs growing interest in managing hereditary forms of cancer, as indicated by the recent acceleration of related research [1,11,20,22,28,29]. This trend aligns with the increasing demand for genetic counseling and testing for hereditary forms of cancer [1,2]. Given the rapid advancements in genetic testing technologies, risk assessment, and treatment options [1,2], there is a need for targeted and up-to-date training programs for HCPs, ensuring they are well-equipped to meet increasing clinical demands in cancer care.

Previous studies exploring the learning needs of HCPs have predominantly focused on managing HBOC-affected families in North America and Europe [1,10,20,21,22,24,25,26,27,28,29,30]. This limited scope of research could hinder our understanding of comprehensive care strategies for families facing diverse hereditary cancer risks and families from different cultural and ethnic backgrounds [8,33]. To address this gap, future studies need to investigate the learning needs of HCPs managing various hereditary cancer syndromes (e.g., Lynch syndrome, Li-Fraumeni syndrome, and Familial Adenomatous Polyposis) and patients from diverse cultural and ethnic backgrounds [5].

In this review, the discussion of the learning needs of HCPs primarily focused on physicians and genetic counselors [11,20,21,24,26,27,28,29,30], while non-physician HCPs (e.g., nurses, psychotherapists, psychologists) and clinical geneticists were underreported [20,21,26,27,30]. This result is not surprising, as physicians mainly order genetic tests and engage in decision-making for cancer risk management, and genetic counselors provide genetic counseling [13,34]. However, genetic care is often delivered by multidisciplinary teams [30,33], and there is a shortage of genetic counselors in certain regions, leading non-genetic professionals such as nurses to provide genetic counseling [30,31]. As part of this context, our study also indicated that primary care providers and nurses sought to clarify their roles in the context of genetic care [20,24]. Thus, to ensure that genetic services are comprehensive, it would be valuable if future studies investigated the learning needs of various HCPs (e.g., nurses, psychotherapists, psychologists, and clinical geneticists).

HCPs emphasized their need for training regarding the effective communication of genetic diagnoses and managing diverse cancer risks for different members of families affected by hereditary cancer [1,11,20,24,25,28,29]. This finding suggests that HCPs face increasing challenges in communicating genetic information and the associated recommendations to patients [35]. To address this, HCPs required practical knowledge and skills, such as understanding genetic test results and their implications, facilitating shared decision-making, and clarifying patient values [1,4,20,24,25,28], rather than focusing on the basic principles of traditional genetic education such as DNA/RNA structure, mutation processes, collecting family information, and drawing pedigrees [13]. Considering that interactive approaches are beneficial in enhancing practical skills [20], future training for HCPs can prioritize more practical and engaging learning strategies, including role-playing/discussions, tool-based exercises (e.g., value clarification methods and cancer prediction models), and simulated patient scenarios, moving beyond traditional lecture formats [11,20].

The findings of this systematic review highlight that learning needs differ according to profession, as the roles of different HCPs vary in the context of genetic cancer care. Specifically, physicians needed information regarding the assessment and management of cancer risk [12,28]; genetic counselors sought guidance for supporting familial issues [10,29]; and nurses sought understanding in the genetic decision-making process and resources for supporting the genetic care system [20]. Non-genetic professionals required a wider range of information than genetic professionals [10,25,29], suggesting they have more extensive learning needs. Our results imply that educational content and strategies should be tailored to each profession’s needs and roles in genetic care. Additionally, since clinical geneticists’ learning needs were underreported, further exploration is necessary. Based on this deeper understanding of learning needs, effective education programs can be developed.

HCPs recognize that families affected by hereditary cancer need a broad spectrum of information, including information related to cancer risk, its management, and related social/psychological/familial issues. This result aligns with the informational needs of members from families affected by hereditary cancer identified in a previous systematic review [36]. However, there is a discrepancy between the information that HCPs believe should be provided and what patients actually find necessary. Based on a prior systematic review, members from families affected by hereditary forms of cancer need more extensive information on lifestyle behaviors, the role of pathogenic variants in carcinogenesis, treatment prognosis, and options for breast reconstruction [36], which HCPs do not fully acknowledge in our review. The discrepancy between the HCP-perceived necessary information and patient-reported information needs, which cover more specific and detailed information [36], may imply a gap between the information provided by HCPs and the real needs of patients. However, future research is needed to confirm these differences. Identifying and fulfilling the gaps between patients’ needs and HCPs’ perceptions could guide effective patient–provider communication.

### Strengths and Limitations

We included studies published in English from January 2013 to April 2024, potentially excluding relevant articles in other languages and earlier timeframes. We explored the perspectives of diverse HCPs, including physicians, nurses, psychotherapists, and genetic counselors, facilitating a deeper understanding of the differences between their learning needs based on their professions. However, since most studies originated from North America and Europe, our findings may not directly apply to HCPs in different regions or those with different healthcare systems, who may have distinct learning needs and perspectives. The findings may limit the generalizability and transferability of the results, since the population of HCPs in the selected studies and the number of reviewed studies were limited, and many studies collected data using qualitative designs.

Nonetheless, this review is novel, as it is the first to synthesize evidence regarding the learning needs of HCPs and their perspectives regarding the essential information to provide to families affected by hereditary cancer. Furthermore, as this study comprehensively and systematically explored the learning needs among HCPs, the developed taxonomy of leaning needs can be used to assess HCPs’ learning needs and develop tailored educational strategies according to profession.

## 5. Conclusions

We identified the learning needs of HCPs and compared them across professions, along with their perspectives on essential information for families affected by hereditary cancer, using thirteen studies involving 332 HCPs. Most studies focused on the learning needs of physicians caring for families affected by Hereditary Breast and Ovarian Cancer in North America and Europe. HCPs required training in practical counseling skills rather than training on the basics of molecular genetics. While HCPs recognized the importance of providing a wide range of information to families, their learning needs varied by profession: physicians needed training in assessing cancer risk and supporting decision-making in risk management; nurses required information on human/material resources and genetic care systems; genetic counselors sought skills in guiding family communication and family planning. The essential information HCPs identified for families focused on risk-reducing strategies, personalized cancer risk assessments, family implications, psychological issues, (cascade) genetic testing, and social concerns. These findings have potential implications for the development of training programs for HCPs, as this taxonomy of learning needs could be used to assess these needs and guide the content and structure of training programs based on profession. HCPs’ views on what constitutes essential information for families affected by hereditary cancer can inform genetic counseling content. Future research should explore the learning needs of non-physician HCPs caring for families from diverse forms of hereditary cancer and diverse cultural backgrounds.

## Figures and Tables

**Table 1:** Summarized characteristics of selected studies (N = 13 studies).

Characteristics of Studies		Number of Studies (%) (N = 13)	References
Publication year	2019–2024	8 (61.5)	[1,11,20,21,22,27,28,29]
	2013–2018	5 (38.5)	[10,24,25,26,30]
Continent	North America	6 (46.2)	[10,21,24,26,28,29]
	Europe	5 (38.5)	[1,20,25,27,30]
	Asia	1 (7.7)	[11]
	Oceania	1 (7.7)	[22]
Number of HCPs	≥101	1 (7.7)	[1]
	51–100	2 (15.4)	[11,22]
	11–50	4 (30.8)	[10,28,29,30]
	1–11	6 (46.2)	[20,21,24,25,26,27]
Study design	Qualitative study	5 (38.5)	[10,25,26,27,28]
	Experimental study	2 (15.4)	[20,29]
	Descriptive study using Delphi method	2 (15.4)	[11,30]
	Descriptive cross-sectional study	2 (15.4)	[1,24]
	Mixed methods research	2 (15.4)	[21,22]
Data collection method	Questionnaire/survey	4 (30.8)	[1,11,24,30]
	Interviews/written feedback/focus group	7 (53.8)	[10,20,25,26,27,28,29]
	Interviews and questionnaire	2 (15.4)	[21,22]
Targeted patients and families of HCPs	Individuals affected by HBOC syndrome	11 (84.6)	[1,10,11,20,21,22,24,26,28,29,30]
	Individuals affected by Lynch syndrome	1 (7.7)	[27]
	Individuals affected by various hereditary cancer ^a^	1 (7.7)	[25]
Research interest ^b^	Cancer risk-reducing strategies	7 (53.8)	[1,20,21,24,27,28,30]
	Performing genetic testing	3 (23.1)	[1,11,20]
	Disclosing genetic test results with family	2 (15.4)	[26,29]
	Needs assessment in cancer risk trajectories	2 (15.4)	[10,22]
	Performing genetic counseling	1 (7.7)	[11]
	Reproductive decision	1 (7.7)	[25]
Characteristics		Number of HCPs (%) (N = 332)	Reference
Numbers of profession in HCPs	Non-genetic professionals	211 (63.6)	[1,11,20,21,24,26,27,28,29,30]
	Physician	205 (61.7)	[1,11,21,24,26,27,28,29,30]
	Nurse	12 (3.6)	[20,21,30]
	Psychotherapist	1 (0.3)	[26]
	Psychologist	1 (0.3)	[27]
	Statistician	1 (0.3)	[27]
	Genetic professionals	99 (29.8)	[10,21,22,25,26,29,30]
	Genetic counselor	86 (25.9)	[10,21,22,29,30]
	Clinical geneticist	13 (3.9)	[25,26,30]
	Non-genetic professional & genetic professional ^c^	13 (3.9)	[22]

^a^ Studies conducted for individuals affected by HBOC, lynch syndrome, familial adenomatous polyposis, retinoblastoma, paraganglioma, and hereditary diffuse gastric cancer. ^b^ Multiple research interests are reported separately for each study. ^c^ Studies reported a mix of HCPs including non-genetic and genetic professionals. Abbreviations: HBOC, Hereditary Breast and Ovarian Cancer; HCPs, Health Care Professionals.

**Table 2:** Learning needs of HCPs and a comparison across professions (N = 9 studies).

	Non-Genetic Professional		Genetic Professional		References
Learning Needs	Physician	Nurse	Genetic Counselor	Clinical Geneticist	
1. Methods for supporting decision-making					[1,20,24,25,28]
Understanding decision needs, psychosocial needs, and coping strategies	●	●	○	○	[1,20,28]
Shared decision-making	●	○	○	○	[1,24]
Methods to clarify values	○	●	○	○	[20]
Decision-making process in their centers	○	●	○	○	[20]
Optimal time to provide aid in making a decision	○	○	○	●	[25]
Factors to consider when recommending risk management strategies	●	○	○	○	[28]
2. Methods for assessing and managing cancer risk					[1,11,20,24]
Prediction model of cancer (re-)occurrence	●	○	○	○	[1,11,24]
Cancer risk-reducing strategies	●	○	○	○	[1,24]
Process of genetic testing	●	○	○	○	[1,11]
Clinical interpretation of genetic testing	●	●	○	○	[1,20]
VUS	●	○	○	○	[1]
3. Resources for supporting hereditary cancer families					[11,20,25,29]
Material resources	●	●	○	●	[11,20,25]
Human resources	●	●	○	○	[11,20]
4. Guidance for supporting familial issues					[10,22,29]
Family communication	○	○	●	○	[10,29]
Disclosing test results	○	○	○	○	[22]
Family planning	○	○	●	○	[10]
Management of familial distress	○	○	●	○	[10]
5. The role of different professions					[20,24]
Primary care provider’s role	●	○	○	○	[24]
Nurse’s role	○	●	○	○	[20]

● represents the learning needs reported by HCPs. ○ represents the learning needs that HCPs did not report.

**Table 3:** Perceptions of HCPs regarding the information that is essential to provide to families affected by hereditary cancer (N = 6 studies).

Essential Information to Provide to Families Affected by Hereditary Cancer	References
1. Cancer risk-reducing strategies	[10,21,22,30]
General information regarding strategies: process and access to screening, surgery, and medication	[10,21,22,30]
Impact of strategies: side effects, cancer risk, body image, breastfeeding, life style, and financial impact	[10,22]
Choosing between risk-reducing strategies	[22]
Insurance coverage for different strategies	[22]
2. Personalized cancer risk	[22,27,30]
Timing of cancer development	[22]
Lifetime cancer risk	[22]
Tailored cancer risk	[27]
Men’s cancer risk according to genetic variant	[30]
3. Familiar implications of hereditary cancer risk	[10,22,30]
Communicating genetic testing results with relatives	[10,22]
Reproductive and family planning issues	[10,22]
Transmitting the pathogenic variant to (grand)children	[22,30]
Transmitting the pathogenic variant to relatives	[30]
4. Psychological issues	[10,22]
Emotional management and coping strategies for patients	[10,22]
Emotional management and coping strategies for family members/relatives	[22]
Affective forecasting	[10]
5. Genetic testing	[10,22]
Benefits and harms of knowing their genetic testing results	[10,22]
Cost and insurance coverage of genetic testing	[22]
Understanding genetic testing results	[22]
Timing of genetic testing	[10]
6. Social issues related to genetic testing	[10,22]
Coping with the stigma associated with a genetic diagnosis	[22]
Disclosing testing results to friends	[22]
Future health insurance	[10]
Social resources	[10]

## Data Availability

The data are available upon reasonable request to the authors.

## Supplementary Materials

The following supporting information can be downloaded at: https://www.mdpi.com/article/10.3390/cancers16111963/s1, Table S1: Study inclusion and exclusion criteria; Table S2: Search terms and strategies according to database; Table S3: List of excluded studies and reasons for exclusion; Table S4: Methodological quality assessment results of included studies; Table S5: Characteristics of included studies.
