# Supplementary material for: Healthcare Professionals’ Learning Needs and Perspectives on Essential Information in Genetic Cancer Care: A Systematic Review
Source: Cancers (Basel). 2024 May 22;16(11):1963. doi: 10.3390/cancers16111963 (PMC11171145; doi:10.3390/cancers16111963)
Supplement: Supplementary file 1 [file cancers-16-01963-s001.zip › cancers-3017012-supplementary.pdf]

Supplementary material

Table legends

Table S1. Study inclusion and exclusion criteria.

Table S2. Search terms and strategies according to database.

Table S3. List of excluded studies and reasons for exclusion.

Table S4. Methodological quality assessment results of included studies.

Table S5. Characteristics of included studies (N=13).

Table S1. Study inclusion and exclusion criteria.

	Inclusions	Exclusions
**Target population**	• Studies on healthcare professionals who treat or counsel or care hereditary cancer patient, their relatives, or non-blood relatives (e.g., spouses, in-laws, etc.).	• Studies that were not conducted with the target population, including the following:<br>- Hereditary cancer patient, their relatives, or non-blood relatives (e.g., spouses, in-laws, etc.)<br>- Animal experiments or pre-clinical experiments.
**Outcomes**	• Studies that reported perspectives and learning needs related to genetic counseling with hereditary cancer patient, their relatives, or non-blood relatives.	• Studies that did not examine perspectives and needs.
**Study design**	• Randomized controlled trial, cross-sectional, case-control, retrospective or prospective cohort studies, qualitative studies, mixed methods research, Delphi research, and case reports.	• Not original study (e.g. reviews, letters, editorials, notes, etc.).
**Publication type**	• Peer-reviewed study.	• Not peer-reviewed study (e.g. conference abstracts).
**Language**	• Written in English.	-

Table S2. Search terms and strategies according to database.

No.	Search strategies	Searched articles		
**Database**		**MEDLINE**	**EMBASE**	**PsycInfo**
1	(*BRCA*1 or *BRCA*2 or RAD51D or PALB2 or ATM or MUTYH or CHEK2 or MLH1 or MSH2 or MSH6 or TP53 or BRIP1).ti,ab.	67,170	112,502	1,098
2	(((hereditary or genetic* or predispos* or famil* or inherite* or mutation*) adj2 (cancer or tumour* or tumor* or tumo?r or neoplastic* or neoplasm* or neoplasia* or malignan*)) or Lynch syndrome or MUTYH associated polyposis or Li-Fraumeni syndrome).ti,ab.	60,309	93,176	1,949
3	((prevent* or risk reducing or prophylact* or chemoprevention) adj2 (cancer or tumour* or tumor* or tumo?r or neoplastic* or neoplasm* or neoplasia* or malignan*)).ti,ab.	44,943	60,701	2,452
4	((DNA or genetic or gene or fertility) adj1 (testing or counsel* or (digital adj1 health*) or application* or apps* or portable software or portable electronic* or smartphone or virtual realit* or instructional virtual intervention)).ti,ab.	56,939	92,555	5,902
5	OR/1-4	208,550	321,570	10,220
6	(cancer predispos* or cancer risk or previvor* or carrier* or survivor* or patient* or famil* or relative* or blood or variant* or proband* or offspring* or child or children or parent* or physician* or (nurs* adj1 (licensur* or advance* or practice* or staff*)) or (health* adj1 (professional* or provider* or personnel* or worker*)) or counsel*).ti,ab.	13,511,220	18,142,5<br>29	2,228,631
7	((decision* or choice* or choose or chosen or chose or decid* or select or prefer) adj2 (support* or making or make* or aid or approach* or content or question* or concern* or worr* or advice* or need* or unmet or met or seek* or fulfil* or gap or satisf* or preference* or prefer* or attitude* or perspective* or perceive* or expectat* or demand*)).ti,ab.	332,907	451,814	169,993
8	5 and 6 and 7	3,981	7,158	953
9	limit 8 to yr="2013 -Current"	**2,809**	**5,539**	**555**
**Database**			**CINAHL**	
1	TI (*BRCA*1 or *BRCA*2 or RAD51D or PALB2 or ATM or MUTYH or CHEK2 or MLH1 or MSH2 or MSH6 or TP53 or BRIP1)			2,624
2	TI (((hereditary or genetic or predispose or familial or inherit or mutation) AND (cancer or tumour or tumor or neoplastic or neoplasm or neoplasia or malignancy or malignant)) or Lynch syndrome or MUTYH associated polyposis or Li-Fraumeni syndrome)			11,520
3	TI ((prevent or risk reducing or prophylact or chemoprevention) AND (cancer or tumour or tumor or neoplastic or neoplasm or neoplasia or malignancy or malignant))			2,103

4	TI ((DNA or genetic or gene or fertility) AND (testing or counsel or (digital AND health) or application or apps or portable software or portable electronic or smartphone or virtual reality or instructional 5virtual intervention))	5,068
5	OR/1-4	19,506
6	TI (cancer predispose or cancer risk or previvor or carrier or survivor or patient or family or relative or blood or variant or proband or offspring or child or children or parent or physician or (nurse AND (licensure or advance or practice or staff)) or (health AND (professional or provider or personnel or worker)) or counseling)	1,475,529
7	TI (decision or choice or choose or chosen or choes or decide or select or prefer or support or aid or approach or content or question or concern or worry or advice or need or unmet or met or seek or fulfil or gap or satisfaction or preference or prefer or attitude or perspective or perceive or expectation or demand)	598,926
8	5 and 6 and 7	482
9	limit 8 to yr="2013 -Current"	**327**
**Database**		**Cochrane Central Register of Controlled Trials**
1	(*BRCA*1 or *BRCA*2 or RAD51D or PALB2 or ATM or MUTYH or CHEK2 or MLH1 or MSH2 or MSH6 or TP53 or BRIP1):ti,ab	2,813
2	(((hereditary or genetic? or predispos? or famil? or inherite? or mutation?) NEAR/2 (cancer or tumour? or tumor? or tumo?r or neoplastic? or neoplasm? or neoplasia? or malignan?)) or Lynch syndrome or MUTYH associated polyposis or Li-Fraumeni syndrome):ti,ab	1,579
3	((prevent? or risk reducing or prophylact? or chemoprevention) NEAR/1 (cancer or tumour? or tumor? or tumo?r or neoplastic? or neoplasm* or neoplasia* or malignan*)):ti,ab	4,058
4	((DNA or genetic or gene or fertility) NEAR/1 (testing or counsel? or (digital NEAR/1 health?) or application? or apps? or portable software or portable electronic? or smartphone or virtual realit? or instructional virtual intervention)):ti,ab	1,609
5	#1 or #2 or #3 or #4	9,199
6	(cancer predispos? or cancer risk or previvor? or carrier? or survivor? or patient? or famil? or relative? or blood or variant? or proband? or offspring? or child or children or parent? or physician? or (nurs? NEAR/1 (licensur? or advance? or practice? or staff?)) or (health? NEAR/1 (professional? or provider? or personnel? or worker?)) or counsel?):ti,ab	1,460,082
7	((decision? or choice? or choose or chosen or choes or decid? or select or prefer) NEAR/2 (support? or making or make? or aid or approach? or content or question? or concern? or worr? or advice? or need? or unmet or met or seek? or fulfil? or gap or satisf? or preference? or prefer? or attitude? or perspective? or perceive? or expectat? or demand?)):ti,ab	23,841

8	#5 and #6 and #7	393
9	limit 8 to yr="2013 -Current"	**299**

Table S3. List of excluded studies and reasons for exclusion.

**Identified studies from databases searching**				
No	1st author	Published year	Title	Reason for exclusion
1	Adam	2019	Assessing an interactive online tool to support parents' genomic testing decisions	Not eligible population
2	Adolph	2022	Hereditary ovarian cancer risk reduction: a retrospective evaluation of patient perspectives and service provision at a regional hereditary gynecologic cancer clinic 2006-2016	Not eligible outcome
3	Ain	2023	Does mainstream *BRCA* testing affect surgical decision-making in newly-diagnosed breast cancer patients?	Not eligible population
4	Albada	2014	Counselee participation in follow-up breast cancer genetic counselling visits and associations with achievement of the preferred role, cognitive outcomes, risk perception alignment and perceived personal control	Not eligible publication type
5	Archer	2022	Personalized risk prediction in hereditary breast and ovarian cancer: a protocol for a multi-centre randomized controlled trial	Not eligible study design
6	Bang	2022	Genetic testing on patients with developmental delay: a preliminary study from the perspective of physicians	Not eligible population
7	Barton	2019	Before Facebook and before social media...we did not know anybody else that had this: parent perspectives on internet and social media use during the pediatric clinical genetic testing process	Not eligible population
8	Battistuzzi	2019	A qualitative study on decision-making about *BRCA*1/2 testing in Italian women	Not eligible population
9	Benedict	2022	The impact of cancer-related financial toxicity on reproductive concerns and family-building decision-making in post-treatment survivorship	Not eligible population
10	Birch	2019	Assessing shared decision-making clinical behaviors among genetic counsellors	Not eligible population
11	Bokkers	2022	Mainstream genetic testing for women with ovarian cancer provides a solid basis for patients to make a well-informed decision about genetic testing	Not eligible population
12	Bond	2023	Disclosure of genetic risk to dating partners among young adults with von Hippel-Lindau disease	Not eligible outcome
13	Braude	2017	An exploration of Australian psychologists' role in assessing women considering risk-reducing or contralateral prophylactic mastectomy	Not eligible population
14	BrookWhite	2018	Genetic testing for hereditary breast cancer: the decision to decline	Not eligible outcome
15	Burcher	2013	Oncology health professionals' attitudes toward treatment-focused genetic testing for women newly diagnosed	Not eligible population

			with breast cancer	
16	Campwala	2019	Personalizing decision-making for patients choosing contralateral prophylactic mastectomy: a utility analysis of genetic factors and the relative risk of breast cancer	Not eligible population
17	Carbonara	2021	A cost decision model supporting treatment strategy selection in *BRCA*1/2 mutation carriers in breast cancer	Not eligible population
18	Casalino	2023	Exploring the role of a multidisciplinary hereditary gynecologic oncology clinic in epithelial ovarian cancer risk-reducing surgical decision-making practices: a mixed-methods study	Not eligible outcome
19	Caskey	2020	Expectations of surveillance for non-*BRCA* gene mutation carriers at increased risk for breast cancer	Not eligible outcome
20	Catania	2016	Improved health perception after genetic counselling for women at high risk of breast and/or ovarian cancer: construction of new questionnaires--an Italian exploratory study	Not eligible population
21	Chan	2017	Reproductive decision-making in women with *BRCA*1/2 mutations	Not eligible population
22	Chang	2022	Differences in willingness to undergo *BRCA*1/2 testing and risk reducing surgery among the general public, cancer patients, and healthcare professionals: a large population-based survey	Not eligible population
23	Chanouha	2023	Healthcare decision makers' perspectives on the creation of new genetic counselor positions in north America: exploring the case for psychiatric genetic counseling	Not eligible population
24	Cherry	2013	Understanding the needs of women considering risk-reducing salpingo-oophorectomy	Not eligible population
25	Chiba	2016	Impact that timing of genetic mutation diagnosis has on surgical decision making and outcome for *BRCA*1/*BRCA*2 mutation carriers with breast cancer	Not eligible study design
26	Chin	2020	Knowledge, awareness, and perception of genetic testing for hereditary disorders among Malaysians in klang valley	Not eligible population
27	Choi	2021	Association of risk-reducing salpingo-oophorectomy with breast cancer risk in women with *BRCA*1 and *BRCA*2 pathogenic variants	Not eligible outcome
28	Christensen	2022	'Never once was i thinking the c-word': parent perspectives on the facilitators and barriers to getting a childhood cancer diagnosis	Not eligible population
29	Collins	2016	I prevent: a tailored, web-based, decision support tool for breast cancer risk assessment and management	Not eligible population
30	Comeaux	2022	Risk-reducing mastectomy decisions among women with mutations in high- and moderate- penetrance breast cancer susceptibility genes	Not eligible outcome

31	Compte	2022	Understanding cancer genetic risk assessment motivations in a remote tailored risk communication and navigation intervention randomized controlled trial	Not eligible population
32	Connors	2014	Decision making after *BRCA* genetic testing. Down the road of transition	Not eligible outcome
33	Copur	2019	Universal genetic testing for all breast cancer patients	Not eligible population
34	Cukier	2013	Factors associated with psychological distress among women of African descent at high risk for *BRCA* mutations	Not eligible population
35	Cullinan	2020	An eHealth decision-support tool to prioritize referral practices for genetic evaluation of patients with Wilms tumor	Not eligible population
36	Dason	2022	Providers' perspectives on the reproductive decision-making of *BRCA*-positive women	Not eligible population
37	Davis	2020	Genetic counselors with advanced skills: ii. A new career trajectory framework	Not eligible population
38	Dean	2018	Men's and women's approaches to disclosure about *BRCA*-related cancer risks and family planning decision-making	Not eligible outcome
39	Dean	2018	Previvors' uncertainty management strategies for hereditary breast and ovarian cancer	Not eligible study design
40	Dean	2022	Feasibility, acceptability, and outcomes of a pilot intervention facilitating communication about family building between patients with inherited cancer risk and their partners	Not eligible outcome
41	Dean	2023	Shared decision-making experiences of couples with inherited cancer risk regarding family building	Not eligible outcome
42	Derks-Smeets	2014	Decision-making on preimplantation genetic diagnosis and prenatal diagnosis: a challenge for couples with hereditary breast and ovarian cancer	Not eligible outcome
43	Dettwyler	2022	Timely cancer genetic counseling and testing for young women with breast cancer: impact on surgical decision-making for contralateral risk-reducing mastectomy	Not eligible study design
44	Dibble	2022	Perceptions and care recommendations from previvors: qualitative analysis of female *BRCA*1/2 mutation carriers' experience with genetic testing and counseling	Not eligible population
45	Dick	2022	Psychological factors and the uptake of preventative measures in *BRCA*1/2 pathogenic variant carriers: results of a prospective cohort study	Not eligible outcome
46	Dilzell	2014	Evaluating the utilization of educational materials in communicating about Lynch syndrome to at-risk relatives	Not eligible population
47	Donenberg	2019	A clinically structured and partnered approach to genetic testing in Trinidadian women with breast cancer and their families	Not eligible outcome

48	Donnelly	2013	Reproductive decision-making in young female carriers of a *BRCA* mutation	Not eligible outcome
49	Eismann	2016	Long-term experiences with genetic consultation in people with hereditary breast and ovarian cancer	Not eligible outcome
50	Emiroglu	2023	Is breast conserving surgery efficacious in breast cancer patients with *BRCA*1 or *BRCA*2 germline mutation?	Not eligible population
51	Erali	2023	Pivotal role of genetic counselors in the uptake of germline genetic testing in non-metastatic breast cancer and the impact of testing on surgical decision making	Not eligible population
52	Fadda	2020	Physicians communicating with women at genetic risk of breast and ovarian cancer: are we in the middle of the ford between contradictory messages and unshared decision making?	Not eligible outcome
53	Fadda	2021	Effect of inquiry-based stress reduction on well-being and views on risk-reducing surgery among women with *BRCA* variants in Israel: a randomized clinical trial	Not eligible outcome
54	Fang	2023	Uptake of genetic testing among patients seeking cancer genetic counseling in Taiwan	Not eligible outcome
55	Fenton	2023	Cancer caregivers' prognostic and end-of-life communication needs and experiences and their impact	Not eligible population
56	Finley	2016	Genetic counseling supervisors' self-efficacy for select clinical supervision competencies	Not eligible population
57	Finn	2023	Motivation and family communication in hereditary prostate cancer genetic testing: survey of patients from a US tertiary medical center	Not eligible population
58	Fischer	2013	Evaluating the performance of the breast cancer genetic risk models Boadicea, ibis, bracero and Claus for predicting *BRCA*1/2 mutation carrier probabilities: a study based on 73not eligible outcome2 families from the German hereditary breast and ovarian cancer consortium	Not eligible outcome
59	Fischer	2017	Psychological and medical need for assistance in women with increased risk for hereditary breast and ovarian cancer-a qualitative analysis	Not eligible publication type
60	Forrest	2021	Finding the five-year window: a qualitative study examining young women's decision-making and experience of using tamoxifen to reduce *BRCA*1/2 breast cancer risk	Not eligible population
61	Foster	2017	Patient understanding of genetic information influences reproductive decision making in retinoblastoma	Not eligible population
62	Frijstein	2023	Effects of a pre-visit online information tool about genetic counselling for ovarian cancer patients, a randomized controlled trial	Not eligible outcome
63	Fuller	2022	Reaction mechanisms, kinetics, and improved catalysts for ammonia synthesis from hierarchical high throughput catalyst design	Not eligible population

64	Gaba	2022	Surgical decision making in premenopausal *BRCA* carriers considering risk-reducing early salpingectomy or salpingo-oophorectomy: a qualitative study	Not eligible population
65	Gaba	2022	Unselected population genetic testing for personalized ovarian cancer risk prediction: a qualitative study using semi-structured interviews	Not eligible population
66	Gamble	2023	Decision-making and regret in patients with germline CDH1 variants undergoing prophylactic total gastrectomy	Not eligible outcome
67	Georgiou	2016	Genetic testing for childhood cancer survivors' risk of late effects: consumer understanding, acceptance and willingness-to-pay	Not eligible population
68	Ghezelayagh	2020	Perceptions of risk and reward in *BRCA*1 and *BRCA*2 mutation carriers choosing salpingectomy for ovarian cancer prevention	Not eligible outcome
69	Gilbert	2017	Differences among a modern cohort of *BRCA* mutation carriers choosing bilateral prophylactic mastectomies compared to breast surveillance	Not eligible population
70	Githaiga	2017	Socio-cultural contexts of end- of- life conversations and decisions: bereaved family cancer caregivers' retrospective co-constructions	Not eligible population
71	Glassey	2018	Influences on decision-making for young women undergoing bilateral prophylactic mastectomy	Not eligible population
72	Gleeson	2013	Communication and information needs of women diagnosed with ovarian cancer regarding treatment-focused genetic testing	Not eligible population
73	Godino	2018	Decision making and experiences of young adults undergoing presymptomatic genetic testing for familial cancer: a longitudinal grounded theory study	Not eligible study design
74	Godino	2021	Genetic counselling as a route to enhanced autonomy: using a sequential mixed methods research approach to develop a theory regarding presymptomatic genetic testing for young adults at risk of inherited cancer syndromes	Not eligible outcome
75	Gore	2023	Performance of the shared decision-making process scale for use in evaluation of hereditary cancer genetic testing decisions	Not eligible outcome
76	Gornick	2018	Knowledge regarding and patterns of genetic testing in patients newly diagnosed with breast cancer participating in the I can decide trial	Not eligible outcome
77	Greenup	2013	Prevalence of *BRCA* mutations among women with triple-negative breast cancer (TNBC) in a genetic counseling cohort	Not eligible outcome

78	Greenup	2013	Impact of race in prevalence of *BRCA* mutations among women with triple-negative breast cancer (TNBC) in a genetic counseling cohort	Not eligible publication type
79	Gregersen	2022	Genetic testing in adult survivors of Retinoblastoma in Denmark: a study of the experience and impact of genetic testing many years after initial diagnosis	Not eligible population
80	Gregersen	2023	Danish heritable Retinoblastoma survivors' perspectives on reproductive choices: "it's important for me, not to pass on this condition"	Not eligible population
81	Gregory	2022	Polygenic risk in familial breast cancer: changing the dynamics of communicating genetic risk	Not eligible population
82	Grimmett	2019	Development of breast cancer choices: a decision support tool for young women with breast cancer deciding whether to have genetic testing for *BRCA*1/2 mutations	Not eligible study design
83	Haddad	2021	Family planning in carriers of *BRCA*1 and *BRCA*2 pathogenic variants	Not eligible population
84	Hallowell	2016	An investigation of the factors effecting high-risk individuals' decision-making about prophylactic total gastrectomy and surveillance for hereditary diffuse gastric cancer (HDGC)	Not eligible population
85	Harding	2022	Health literacy in communication, decision-making and outcomes among cancer patients, their families and clinicians in India: a multicentre cross-sectional qualitative study	Not eligible population
86	Harmsen	2018	A patient decision aid for risk-reducing surgery in premenopausal *BRCA*1/2 mutation carriers: development process and pilot testing	Not eligible population
87	Hasser	2023	Measuring high-risk parents' opinions about direct-to-consumer genetic testing for adult-onset inherited cancer syndromes in their adolescent and young adult children	Not eligible outcome
88	Hasson	2020	Comparison of patient susceptibility genes across breast cancer: implications for prognosis and therapeutic outcomes	Not eligible outcome
89	Hawley	2014	Social and clinical determinants of contralateral prophylactic mastectomy	Not eligible population
90	Herold	2024	Implementing mainstream genetic counseling within the area-wide network of the German Consortium Hereditary Breast and Ovarian Cancer (GC-HBOC): Satisfaction of primary care providers with the provided state-of-the-art training by the Cologne Center	Not eligible outcome
91	Henderson	2021	Development of a culturally sensitive narrative intervention to promote genetic counseling among African American women at risk for hereditary breast cancer	Not eligible study design
92	Henderson	2022	Pilot study of a culturally sensitive intervention to promote genetic counseling for breast cancer risk	Not eligible outcome

93	Henneman	2020	Do preferred risk formats lead to better understanding? A multicenter controlled trial on communicating familial breast cancer risks using different risk formats	Not eligible population
94	Henneman	2021	Family identity and roles in the context of Li-Fraumeni syndrome: "no one's like us mutants"	Not eligible outcome
95	Hesse-Biber	2014	The genetic testing experience of *BRCA*-positive women: deciding between surveillance and surgery	Not eligible population
96	Hesse-Biber	2017	Within-gender differences in medical decision making among male carriers of the *BRCA* genetic mutation for hereditary breast cancer	Not eligible outcome
97	Hickey	2020	What information do healthcare professionals need to inform premenopausal women about risk-reducing salpingo-oophorectomy?	Not eligible population
98	Himes	2019	Does family communication matter? Exploring knowledge of breast cancer genetics in cancer families	Not eligible population
99	Hoberg-Vetti	2019	Cancer-related distress in unselected women with newly diagnosed breast or ovarian cancer undergoing *BRCA*1/2 testing without pretest genetic counseling	Not eligible outcome
100	Hooker	2014	Long-term satisfaction and quality of life following risk reducing surgery in *BRCA*1/2 mutation carriers	Not eligible study design
101	Horowitz	2016	Determining the effects and challenges of incorporating genetic testing into primary care management of hypertensive patients with African ancestry	Not eligible population
102	Hoskins	2014	In their own words: treating very young *BRCA*1/2 mutation-positive women with care and caution	Not eligible population
103	Hovick	2019	Understanding *BRCA* mutation carriers' preferences for communication of genetic modifiers of breast cancer risk	Not eligible outcome
104	Hynes	2020	Group plus "mini" individual pre-test genetic counselling sessions for hereditary cancer shorten provider time and improve patient satisfaction	Not eligible outcome
105	Interrante	2017	Randomized non inferiority trial of telephone vs in-person genetic counseling for hereditary breast and ovarian cancer: a 12-month follow-up	Not eligible outcome
106	Isselhard	2020	Implementation and evaluation of a nurse-led decision-coaching program for healthy breast cancer susceptibility gene (*BRCA*1/2) mutation carriers: a study protocol for the randomized controlled EDCP-*BRCA* study	Not eligible study design
107	Isselhard	2021	The fate of unifocal versus multifocal low-grade dysplasia at the time of colonoscopy in patients with IBD	Not eligible study design
108	Isselhard	2023	Psychological distress and decision-making factors for prophylactic bilateral mastectomy in cancer-unaffected *BRCA*1/2 pathogenic variant carriers	Not eligible population
109	Isselhard	2023	Coping self-efficacy and its relationship with psychological morbidity after genetic test result disclosure: results	Not eligible population

			from cancer-unaffected *BRCA*1/2 mutation carriers	
110	Jackson	2014	Guidance for patients considering direct-to-consumer genetic testing and health professionals involved in their care: development of a practical decision tool	Not eligible population
111	Jacobs	2016	Patient and genetic counselor perceptions of in-person versus telephone genetic counseling for hereditary breast/ovarian cancer	Not eligible population
112	Jacobson	2021	Factors affecting surgical decision-making in carriers of *BRCA*1/2 pathogenic variants undergoing risk-reducing surgery at a dedicated hereditary ovarian cancer clinic	Not eligible outcome
113	Jeffers	2014	Maximizing survival: the main concern of women with hereditary breast and ovarian cancer who undergo genetic testing for *BRCA*1/2	Not eligible population
114	Jennings	2022	Information needs of women undergoing gynecological risk reduction surgery: applying patient-reported findings to improve service delivery	Not eligible population
115	Jones	2021	Perceptions of racially and ethnically diverse women at high risk of breast cancer regarding the use of a web-based decision aid for chemoprevention: qualitative study nested within a randomized controlled trial	Not eligible population
116	Jones	2021	Identification of factors that influence the decision to take chemoprevention in patients with a significant family history of breast cancer: results from a patient questionnaire survey	Not eligible outcome
117	Kalamo	2020	Factors associated with decision-making on prophylactic hysterectomy and attitudes towards gynecological surveillance among women with Lynch syndrome (LS): a descriptive study	Not eligible outcome
118	Kanga-Parabia	2018	Discussions about predictive genetic testing for Lynch syndrome: the role of health professionals and families in decisions to decline	Not eligible outcome
119	Kasting	2019	A randomized controlled intervention to promote readiness to genetic counseling for breast cancer survivors	Not eligible population
120	Katapodi	2013	Individual and family characteristics associated with *BRCA*1/2 genetic testing in high-risk families	Not eligible population
121	Kaur	2022	The risk-reducing effect of aspirin in Lynch syndrome carriers: development and evaluation of an educational leaflet	Not eligible outcome
122	Kautz-Freimuth	2021	Development of decision aids for female *BRCA*1 and *BRCA*2 mutation carriers in Germany to support preference-sensitive decision-making	Not eligible study design
123	Kautz-Freimuth	2022	Evaluation of two evidence-based decision aids for female *BRCA*1/2 mutation carriers in Germany: study protocol for a randomized controlled parallel-group trial	Not eligible study design

124	Kim	2016	Uptake of risk-reducing salpingo-oophorectomy among female *BRCA* mutation carriers: experience at the national cancer center of Korea	Not eligible population
125	Kim	2021	Using a tailored digital health intervention for family communication and cascade genetic testing in Swiss and Korean families with hereditary breast and ovarian cancer: protocol for the dialogue study	Not eligible population
126	Kim	2022	How do patients value the benefit of minimally invasive surgery in cancer treatment?	Not eligible population
127	King	2013	Intentions for bilateral mastectomy among newly diagnosed breast cancer patients	Not eligible population
128	Kinnaird	2021	A prostate cancer risk calculator (PCRC-MRI): use of clinical and magnetic resonance imaging data to predict biopsy outcome in North American men	Not eligible outcome
129	Kinney	2014	Expanding access to *BRCA*1/2 genetic counseling with telephone delivery: a cluster randomized trial	Not eligible outcome
130	Kne	2017	Why is cancer genetic counseling underutilized by women identified as at risk for hereditary breast cancer? Patient perceptions of barriers following a referral letter	Not eligible study design
131	Kotsopoulos	2019	Oophorectomy and risk of contralateral breast cancer among *BRCA*1 and *BRCA*2 mutation carriers	Not eligible outcome
132	Kukafka	2022	Patient and clinician decision support to increase genetic counseling for hereditary breast and ovarian cancer syndrome in primary care: a cluster randomized clinical trial	Not eligible population
133	Lacey	2022	The role of emotional sensitivity to probability in the decision to choose genetic testing	Not eligible population
134	Ladd	2020	Predictors of genetic testing uptake in newly diagnosed breast cancer patients	Not eligible population
135	Lafreniere	2013	Family communication following *BRCA*1/2 genetic testing: a close look at the process	Not eligible population
136	Lenhart	2023	Entrustment decision-making in genetic counseling supervision: exploring supervisor and student perspectives to enhance training practices	Not eligible population
137	Li	2018	Factors influencing the decision to share cancer genetic results among family members: an in-depth interview study of women in an Asian setting	Not eligible population
138	Li	2022	Considerations in methods and timing for delivery of genetic counseling information to pediatric oncology patients and families	Not eligible population
139	Liede	2017	Preferences for breast cancer risk reduction among *BRCA*1/*BRCA*2 mutation carriers: a discrete-choice experiment	Not eligible population
140	Lin	2020	Feasibility and acceptability of a culturally adapted advance care planning intervention for people living with advanced cancer and their families: a mixed methods study	Not eligible population

141	Lloyd	2022	Barriers and facilitators to using aspirin for preventive therapy: a qualitative study exploring the views and experiences of people with Lynch syndrome and healthcare providers	Not eligible population
142	Lynce	2021	*BRCA*1/2 mutations and risk-reducing bilateral salpingo-oophorectomy among Latinas: the uptake study	Not eligible outcome
143	Macadam	2021	Prophylactic surgery in the *BRCA*+ patient: do women develop breast cancer while waiting?	Not eligible outcome
144	Machirori	2019	Black and minority Ethnic women's decision-making for risk reduction strategies after *BRCA* testing: use of context and knowledge	Not eligible population
145	Maitra	2021	Genetic counseling clinic at aims (New Delhi)	Not eligible publication type
146	Maksimenko	2022	Effectiveness of secondary risk-reducing strategies in patients with unilateral breast cancer with pathogenic variants of *BRCA*1 and *BRCA*2 subjected to breast-conserving surgery: evidence-based simulation study	Not eligible population
147	Manne	2019	Decisional conflict among breast cancer patients considering contralateral prophylactic mastectomy	Not eligible population
148	Manoukian	2019	Risk-reducing surgery in *BRCA*1/*BRCA*2 mutation carriers: are there factors associated with the choice?	Not eligible population
149	Martina	2022	Advance care planning for patients with cancer and family caregivers in Indonesia: a qualitative study	Not eligible study design
150	Matsukawa	2022	Japanese women's reasons for accompaniment status to hereditary breast and ovarian cancer-focused genetic counseling	Not eligible population
151	McGuinness	2022	Strategies to identify and recruit women at high risk for breast cancer to a randomized controlled trial of web-based decision support tools	Not eligible outcome
152	McVeigh	2023	A pilot study investigating feasibility of mainstreaming germline *BRCA*1 and *BRCA*2 testing in high-risk patients with breast and/or ovarian cancer in three tertiary cancer centres in Ireland	Not eligible population
153	Meadows	2018	Distinctive psychological and social experiences of women choosing prophylactic oophorectomy for cancer prevention	Not eligible outcome
154	Medendorp	2020	'We don't know for sure': discussion of uncertainty concerning multigene panel testing during initial cancer genetic consultations	Not eligible population
155	Meiser	2013	Psychosocial factors and uptake of risk-reducing salpingo-oophorectomy in women at high risk for ovarian cancer	Not eligible outcome
156	Meiser	2016	When knowledge of a heritable gene mutation comes out of the blue: treatment-focused genetic testing in women newly diagnosed with breast cancer	Not eligible study design

157	Meiser	2018	Psychological outcomes and surgical decisions after genetic testing in women newly diagnosed with breast cancer with and without a family history	Not eligible population
158	Metcalfe	2017	Effect of decision aid for breast cancer prevention on decisional conflict in women with a *BRCA*1 or *BRCA*2 mutation: a multisite, randomized, controlled trial	Not eligible population
159	Metcalfe	2021	Rapid genetic testing for *BRCA*1 and *BRCA*2 mutations at the time of breast cancer diagnosis: an observational study	Not eligible population
160	Michalowska	2020	Psychosocial context of the decision-making process and the consequences of preventive procedures in a patient with *BRCA*1 genetic mutation	Not eligible outcome
161	Michalowska	2021	The use of heuristics in genetic testing decision-making: a qualitative interview study	Not eligible population
162	Mochiki	2023	Psychological characteristics of Japanese patients and their family members receiving genetic counseling: a single-institute exploratory study	Not eligible population
163	Morand	2022	Factors impacting adolescent and young adult cancer patients' decision to pursue genetic counseling and testing	Not eligible study design
164	Myklebust	2016	Experience of Norwegian female *BRCA*1 and *BRCA*2 mutation-carrying participants in educational support groups: a qualitative study	Not eligible study design
165	Nagashima	2023	Risk-reducing salpingo-oophorectomy for Japanese women with hereditary breast and ovarian cancer: a single-institution 10-year experience	Not eligible outcome
166	Napoli	2020	Factors that impact risk management decisions among women with pathogenic variants in moderate penetrance genes associated with hereditary breast cancer	Not eligible outcome
167	Ochoa	2022	What happens after referral? Completion rates of genetic counseling evaluations in breast cancer patients	Not eligible population
168	Odejide	2022	Patient, family, and clinician perspectives on location of death for adolescents and young adults with cancer	Not eligible population
169	Olejniczak	2016	Acceptance of, inclination for, and barriers in genetic testing for gene mutations that increase the risk of breast and ovarian cancers among female residents of Warsaw	Not eligible population
170	Oseledchyk	2021	Surgical ovarian suppression for adjuvant treatment in hormone receptor positive breast cancer in premenopausal patients	Not eligible outcome
171	Ozanne	2014	Development of a personalized decision aid for breast cancer risk reduction and management	Not eligible population
172	Padamsee	2023	Racial differences in prevention decision making among U.S. Women at high risk of breast cancer: a qualitative study	Not eligible population

173	Park	2018	Genetic diagnosis before surgery has an impact on surgical decision in *BRCA* mutation carriers with breast cancer	Not eligible population
174	Patenaude	2013	Talking to children about maternal *BRCA*1/2 genetic test results: a qualitative study of parental perceptions and advice	Not eligible population
175	Pederson	2018	Impact of an embedded genetic counselor on breast cancer treatment	Not eligible population
176	Pesce	2021	Patient-reported outcomes among women with unilateral breast cancer undergoing breast conservation versus single or double mastectomy	Not eligible population
177	Pollard	2023	Development and early-stage evaluation of a patient portal to enhance familial communication about hereditary cancer susceptibility testing: a patient-driven approach	Not eligible population
178	Pollard	2023	Development and early-stage evaluation of a patient portal to enhance familial communication about hereditary cancer susceptibility testing: a patient-driven approach	Duplicate with selected article
179	Puski	2018	Involvement and influence of healthcare providers, family members, and other mutation carriers in the cancer risk management decision-making process of *BRCA*1 and *BRCA*2 mutation carriers	Not eligible study design
180	Qureshi	2021	Improving primary care identification of familial breast cancer risk using proactive invitation and decision support	Not eligible outcome
181	Radhakrishnan	2019	Provider involvement in care during initial cancer treatment and patient preferences for provider roles after initial treatment	Not eligible population
182	Rauscher	2017	Take your time, then follow your heart: previvors' advice for communicating about family planning after testing positive for a *BRCA* genetic variant	Not eligible study design
183	Rauscher	2018	I am uncertain about what my uncertainty even is: men's uncertainty and information management of their *BRCA*-related cancer risks	Not eligible population
184	Reumkens	2019	Exploring the preferences of involved health professionals regarding the implementation of an online decision aid to support couples during reproductive decision-making in hereditary cancer: a mixed methods approach	Not eligible population
185	Reumkens	2019	Online decision support for persons having a genetic predisposition to cancer and their partners during reproductive decision-making	Not eligible population
186	Reumkens	2019	The development of an online decision aid to support persons having a genetic predisposition to cancer and their partners during reproductive decision-making: a usability and pilot study	Not eligible outcome
187	Reumkens	2021	Reproductive decision-making in the context of hereditary cancer: the effects of an online decision aid on	Not eligible outcome

			informed decision-making	
188	Rising	2022	Family communication challenges of adolescents and young adults with Li-Fraumeni syndrome: implications for psychosocial care	Not eligible population
189	Rocca	2020	Difficult decisions in women at high genetic risk for cancer: toward an individualized approach	Not eligible study design
190	Rocca	2021	Genetic counseling and testing in African American patients with breast cancer: a nationwide survey of US breast oncologists	Not eligible population
191	Rosa	2022	Development, implementation and initial results of CDSS recommendations for patients at risk of hereditary breast cancer	Not eligible outcome
192	Rosenberg	2013	Perceptions, knowledge, and satisfaction with contralateral prophylactic mastectomy among young women with breast cancer: a cross-sectional survey	Not eligible population
193	Rosenberg	2016	*BRCA*1 and *BRCA*2 mutation testing in young women with breast cancer	Not eligible outcome
194	Rowland	2016	Preparing young people for future decision-making about cancer risk in families affected or at risk from hereditary breast cancer: a qualitative interview study	Not eligible population
195	Sa'at	2022	Decision-making for risk-reducing salpingo-oophorectomy (RRSO) in Southeast Asian *BRCA* mutation carriers with breast cancer: a qualitative study	Not eligible outcome
196	Santerre-Theil	2014	Development of a decision aid to help parents carrying a *BRCA*1/2 mutation make decisions about communication of genetic information to their children	Not eligible population
197	Saule	2018	Risk of serous endometrial carcinoma in women with pathogenic *BRCA*1/2 variant after risk-reducing salpingo-oophorectomy	Not eligible outcome
198	Scherr	2021	How patients deal with an ambiguous medical test: decision-making after genetic testing	Not eligible population
199	Schulman-Green	2020	Patient and family caregiver considerations when selecting early breast cancer treatment: implications for clinical pathway development	Not eligible population
200	Scott	2023	A balancing act: Non-directive communication, risk perceptions, and meeting patient needs in genetic counseling: A South African case study	Not eligible outcome
201	Seenandan-Sookdeo	2016	Parental decision making regarding the disclosure or nondisclosure of a mutation-positive *BRCA*1/2 test result to minors	Not eligible outcome
202	Seven	2022	From probands to relatives: communication of genetic risk for hereditary breast-ovarian cancer and its influence	Not eligible population

			on subsequent testing	
203	Shannon	2024	Development of an Electronic Decision Aid Tool to Facilitate Mainstream Genetic Testing in Ovarian Cancer Patients	Not eligible outcome
204	Sherman	2013	Identifying cognitive and affective profiles of women undergoing *BRCA*1/2 genetic testing using cluster analytic techniques	Not eligible publication type
205	Sherman	2017	Facilitating decision-making in women undergoing genetic testing for hereditary breast cancer: Breconda randomized controlled trial results	Not eligible population
206	Shilling	2020	Using patient perspectives to inform communication training materials for health care professionals discussing *BRCA* mutation testing	Not eligible population
207	Shin	2017	Attitudes toward family involvement in cancer treatment decision making: the perspectives of patients, family caregivers, and their oncologists	Not eligible population
208	Sie	2014	More breast cancer patients prefer *BRCA*-mutation testing without prior face-to-face genetic counseling	Not eligible population
209	Silverman	2018	An applied framework in support of shared decision making about *BRCA* genetic testing	Not eligible population
210	Singh	2013	Impact of family history on choosing risk-reducing surgery among *BRCA* mutation carriers	Not eligible outcome
211	Skrovanek	2021	The emotional states associated with reproductive decision-making in women with a *BRCA* pathogenic variant	Not eligible outcome
212	Steenbeek	2021	Evaluation of a patient decision aid for *BRCA*1/2 pathogenic variant carriers choosing an ovarian cancer prevention strategy	Not eligible population
213	Straw	2022	Acceptability, appropriateness and feasibility of the Latin American and Caribbean code against cancer: perceptions of decision-makers and health professionals in Argentina	Not eligible population
214	Sun	2020	Clinical usefulness of genetic testing for drug toxicity in cancer care: decision-makers' framing, knowledge and perceptions	Not eligible population
215	Sun	2021	Contralateral prophylactic mastectomy and implications for breast reconstruction	Not eligible outcome
216	Tan	2014	Knowledge, attitudes and referral patterns of Lynch syndrome: a survey of clinicians in Australia	Not eligible population
217	Tang	2021	Surgery decision conflict and its related factors among newly diagnosed early breast cancer patients in china: a cross-sectional study	Not eligible population
218	Tercyak	2013	Decisional outcomes of maternal disclosure of *BRCA*1/2 genetic test results to children	Not eligible outcome

219	Tercyak	2023	Results of a randomized controlled trial of a decision support intervention for disclosing maternal *BRCA* genetic test results to children and adolescents	Not eligible population
220	terStege	2022	Development of a patient decision aid for patients with breast cancer who consider immediate breast reconstruction after mastectomy	Not eligible population
221	Tesson	2016	Women's preferences for contralateral prophylactic mastectomy: an investigation using protection motivation theory	Not eligible outcome
222	Tezak	2021	Using an anthropological lens to explore motivators and challenges for follow-up care decision making among female *BRCA*1/2 carriers at risk for inherited cancer	Not eligible study design
223	Tiernan	2022	What would i do? Perspectives on the factors underlying Lynch syndrome genetic testing and results sharing decisions for high-risk colorectal cancer patients	Not eligible population
224	Toguri	2020	Views of advanced cancer patients, families, and oncologists on initiating and engaging in advance care planning: a qualitative study	Not eligible population
225	Tutty	2023	Becoming and being a parent with an inherited predisposition to diffuse gastric cancer: a qualitative study of young adults with a CDH1 pathogenic variant	Not eligible outcome
226	Underhill	2014	Seeking balance: decision support needs of women without cancer and a deleterious *BRCA*1 or *BRCA*2 mutation	Not eligible population
227	Vadaparampil	2014	From observation to intervention: development of a psychoeducational intervention to increase uptake of *BRCA* genetic counseling among high-risk breast cancer survivors	Not eligible population
228	vanderGiessen	2021	Development of a plain-language guide for discussing breast cancer genetic counseling and testing with patients with limited health literacy	Not eligible population
229	vanDriel	2016	Psychological factors associated with the intention to choose for risk-reducing mastectomy in family cancer clinic attendees	Not eligible outcome
230	vanEgdom	2019	Patient-reported outcome measures may optimize shared decision-making for cancer risk management in *BRCA* mutation carriers	Not eligible population
231	Verdial	2021	Genetic testing and surgical treatment after breast cancer diagnosis: results from a national online cohort	Not eligible outcome
232	Villegas	2019	Access to genetic counselors in the Southern United States	Not eligible population
233	Vogel	2013	Development and pilot of an advance care planning website for women with ovarian cancer: a randomized controlled trial	Not eligible population

234	Wang	2020	Treatment decision-making, family influences, and cultural influences of Chinese breast cancer survivors: a qualitative study using an expressive writing method	Not eligible population
235	Wang	2022	Purine nucleoside analogs plus rituximab are an effective treatment choice for hairy cell leukemia-variant	Not eligible outcome
236	Wellman	2023	Where do i go? Who do i go to?: *BRCA* previvors, genetic counselors and family planning	Not eligible outcome
237	Wevers	2017	Rapid genetic counseling and testing in newly diagnosed breast cancer: patients' and health professionals' attitudes, experiences, and evaluation of effects on treatment decision making	Not eligible population
238	Wilkes	2017	Increasing confidence and changing behaviors in primary care providers engaged in genetic counselling	Not eligible population
239	Woldemariam	2021	Breaking bad news in cancer care: Ethiopian patients want more information than what family and the public want them to have	Not eligible population
240	Woo	2021	Preoperative diagnosis of *BRCA*1/2 mutation impacts decision-making for risk-reducing mastectomy in breast cancer patients	Not eligible population
241	Wurtmann	2022	An electronic health record tool increases genetic counseling referral of individuals at hereditary cancer risk: an intervention study	Not eligible population
242	Yadav	2017	Preoperative genetic testing impacts surgical decision making in *BRCA* mutation carriers with breast cancer: a retrospective cohort analysis	Not eligible outcome
243	Yadav	2018	Impact of preoperative *BRCA*1/2 testing on surgical decision making in patients with newly diagnosed breast cancer	Not eligible population
244	Yagil	2022	Family members' experiences of the return to work of cancer survivors	Not eligible population
245	Yang	2022	Machine learning application in personalized lung cancer recurrence and survivability prediction	Not eligible population
246	Yang	2022	Prospective validation of the Boadicea multifactorial breast cancer risk prediction model in a large prospective cohort study	Not eligible outcome
247	Yuen	2020	An in-depth exploration of the post-test informational needs of *BRCA*1 and *BRCA*2 pathogenic variant carriers in Asia	Not eligible population
248	Zigman	2020	Defining health-related quality of life in localized and advanced stages of breast cancer - the first step towards hereditary cancer genetic counseling	Not eligible outcome
249	Zimmermann	2021	Autonomy and social influence in predictive genetic testing decision-making: a qualitative interview study	Not eligible population

Manual searching				
1	Visser	2016	Peer support and additional information in group medical consultations (GMCS) for *BRCA*1/2 mutation carriers: a randomized controlled trial	Not eligible population
2	Modaffari	2019	Concerns and expectations of risk-reducing surgery in women with hereditary breast and ovarian cancer syndrome	Not eligible population
3	Dean	2017	"when information is not enough": a model for understanding *BRCA*-positive previvors' information needs regarding hereditary breast and ovarian cancer risk	Not eligible population
4	Peshkin	2021	The genetic education for men (GEM) trial: development of web-based education for untested men in *BRCA*1/2-positive families	Not eligible population
5	Bredart	2021	Information needs on breast cancer genetic and non-genetic risk factors in relatives of women with a *BRCA*1/2 or PALB2 pathogenic variant	Not eligible population
6	Sa'atH	2021	The needs of Southeast Asian *BRCA* mutation carriers considering risk-reducing salpingo-oophorectomy: a qualitative study	Not eligible population

Table S4. Methodological quality assessment results of included studies.

1st author, published year	**Qualitative study**				
	Q 1.1	Q 1.2	Q 1.3	Q 1.4	Q 1.5
Pollard, 2023	Yes	Yes	Yes	Yes	Yes
Vanderwal, 2023	Yes	Yes	Yes	Yes	Yes
Berger-Hoger, 2022	Yes	Yes	Yes	Yes	Yes
Morton, 2022	Yes	Yes	Yes	Yes	Yes
Reumkens, 2018	Yes	Yes	Yes	Yes	Yes
Santerre-Theil, 2018	Yes	Yes	Yes	Can't tell	Yes
Evans, 2016	Yes	Yes	Yes	Yes	Yes
1st author, publication year	**Quantitative descriptive cross-sectional study**				
	Q 2.1	Q 2.2	Q 2.3	Q 2.4	Q 2.5
Lee, 2022	Yes	Yes	Yes	Yes	Yes

Dick, 2021	Yes	Yes	Yes	Can’t tell	Yes
Jacobs, 2017	Yes	Yes	Yes	Yes	Yes
Rupert, 2013	Yes	Yes	Yes	Can't tell	Yes
1st author, publication year	**Mixed-methods study**				
	Q 3.1	Q 3.2	Q 3.3	Q 3.4	Q 3.5
Young, 2019	Yes	Yes	Yes	Yes	Yes
Jabaley, 2020	Yes	Yes	Can’t tell	Can’t tell	Yes

The quality assessment questions for each item are as follows:

Q 1.1. Is the qualitative approach appropriate to answer the research question?

Q 1.2. Are the qualitative data collection methods adequate to address the research question?

Q 1.3. Are the findings adequately derived from the data?

Q 1.4. Is the interpretation of results sufficiently substantiated by data?

Q 1.5. Is there coherence between qualitative data sources, collection, analysis and interpretation?

Q 2.1. Is the sampling strategy relevant to address the research question?

Q 2.2. Is the sample representative of the target population?

Q 2.3. Are the measurements appropriate?

Q 2.4. Is the risk of nonresponse bias low?

Q 2.5. Is the statistical analysis appropriate to answer the research question?

Q 3.1. Is there an adequate rationale for using a mixed-methods design to address the research question?

Q 3.2. Are the different components of the study effectively integrated to answer the research question?

Q 3.3. Are the outputs of the integration of qualitative and quantitative components adequately interpreted?

Q 3.4. Are divergences and inconsistencies between quantitative and qualitative results adequately addressed?

Q 3.5. Do the different components of the study adhere to the quality criteria of each tradition of the methods involved?

Table S5. Characteristics of included studies (N = 13).

1st author (published year), country	Study design (data collection method)	Number of participants	Study aim	Targeted patients and families of HCPs	Research interest
Pollard (2023), Canada	Experimental study (individual interviews)	13 HCPs<br>- 5 oncologists<br>- 8 genetic counselors	To development and evaluation of a patient portal to enhance familial communication about genetic testing.	Individuals affected by HBOC	Family communication in disclosing genetic test results with family
Vanderwal (2023), USA	Qualitative study (focus group and individual interviews)	15 HCPs<br>- 15 breast surgeons	To identify factors influencing breast surgeon's management recommendations for women with pathogenic variants in moderate penetrance breast cancer susceptibility genes.	Individuals affected by HBOC<br><br>(women with pathogenic variants of *ATM*, *CHEK2*, *NBN*, *CDH*, and *NF1*)	Cancer risk−reducing strategies
Berger-Hoger (2022), Germany	Experimental study (individual interviews and field note)	6 HCPs<br>- 6 oncology nurses	To adapt and pilot nurse-led decision coaching program for healthy *BRCA* mutation carriers.	Individuals affected by HBOC<br><br>(healthy *BRCA1/2* gene mutation carriers)	Cancer risk−reducing strategies and genetic testing
Lee (2022), Malaysia	Descriptive study using Delphi method (questionnaire)	53 HCPs<br>- 21 oncologists<br>- 32 surgeons	To evaluate the attitudes, considerations, and training needs of oncologists and surgeons in mainstreaming<br><br>breast cancer genetic counseling	Individuals affected by HBOC	Mainstreaming genetic counseling and genetic testing
Morton (2022), UK	Experimental study (written feedback from HCPs for developing the template)	6 HCPs<br>- 4 gynecologists<br>- 1 statistician<br>- 1 psychologist	To develop decision aid template which can be adapted for different predispositions to cancer.	Individuals affected by Lynch syndrome	Cancer risk−reducing strategies
Dick (2021), Germany	Descriptive cross-sectional study (questionnaire)	111 HCPs<br>- 111 gynecologists	To assess physician's knowledge and training needs in the field of genetic counseling and testing.	Individuals affected by HBOC<br><br>(*BRCA1*/2 carriers)	Cancer risk−reducing strategies and genetic testing
Jabaley (2020), USA	Experimental study (questionnaire and written feedback from HCPs)	8 HCPs<br>- 3 genetic counselors<br>- 1 physician<br>- 4 advanced practice nurses specializing in	To develop and pilot test a decision aid to facilitate shared<br><br>decision making.	Individuals affected by HBOC<br><br>(unaffected women with a *BRCA1*/2 mutation)	Cancer risk−reducing strategies

		the genomics of breast and ovarian cancer			
Young (2019), Australia	Mixed-methods study (questionnaire and focus group/individual interview)	72 HCPs<br>- 59 genetic counselors<br>- 13 geneticists, nurses, oncologists, psychologists/psychiatrists	To describe the perspectives of at-risk *BRCA* young adult and their preferences for the content and format of information resources.	Individuals affected by HBOC<br><br>(18- to 40-year old adults with a *BRCA1*/2 mutation)	Informational needs
Reumkens (2018), Netherlands	Qualitative study (individual interview)	8 HCPs<br>- 8 clinical geneticists	To assess the needs towards the development of a patient decision aid for reproductive decision-making among couples at risk for hereditary cancer.	Individuals affected by HBOC, lynch syndrome, familial adenomatous polyposis, retinoblastoma, paraganglioma, and hereditary diffuse gastric cancer	Reproductive decision
Santerre-Theil (2018), Canada	Qualitative study<br><br>(written feedback from HCPs)	3 HCPs<br>- 1 geneticist<br>- 1 physician<br>- 1 psychotherapist	To develop a tool to guide parents carrying a *BRCA*1/2 mutation share genetic results with their children.	Individuals affected by HBOC<br><br>(*BRCA*1/2 mutation carriers)	Disclosing genetic test results with underage children
Jacobs (2017), UK	Descriptive study using Delphi method (questionnaires)	16 HCPs<br>- 4 clinical geneticists<br>- 4 genetic counselors<br>- 2 nurse specialists<br>- 2 gynecologists<br>- 2 breast surgeons<br>- 2 clinical oncologists	To investigate areas of agreement and disagreement between HCPs and service users about the messages required by affected women about *BRCA*1/2 and the timing of communicating key messages.	Individuals affected by HBOC<br><br>(women with *BRCA*1/2 mutation and breast or ovarian cancer)	Cancer risk–reducing strategies<br><br>Family implementation
Evans (2016), USA	Qualitative study (individual interview)	12 HCPs<br>- 12 cancer genetic counselors	To identify perceptions of need regarding their cancer risk management among patients and counselors, and to assess the initial acceptability of intervention content, format and delivery mode.	Individuals affected by HBOC<br><br>(young women from HBOC families)	Needs at cancer risk trajectories
Rupert (2013), USA	Descriptive cross-sectional study (questionnaires)	9 HCPs<br>- most were physicians	To create an online tool for identifying and counseling women at increased risk for HBOC in primary care settings, and to conduct a pilot evaluation of the tool.	Individuals affected by HBOC<br><br>(women at increased risk for HBOC)	Cancer risk–reducing strategies

Abbreviations: HBOC, Hereditary Breast and Ovarian Cancer; HCPs, Health Care Professionals
